# Special issue on the battle against complex virus world in the human brain: seizure as a result of viral infection

**DOI:** 10.1186/s42494-021-00049-x

**Published:** 2021-07-16

**Authors:** Weijia Jiang, Jinmei Li, Dong Zhou, Jie Mu

**Affiliations:** 1 West China Medical Publishers, West China Hospital of Sichuan University, 14# Third Section, Renmin Nan Road, Chengdu, 610041 China; 2 Neurology Department, West China Hospital of Sichuan University, 14# Third Section, Renmin Nan Road, Chengdu, 610041 China

*Viruses are the smallest living things known to science, yet they hold the entire planet in their way.*

- from *A Planet of Viruses,* Carl Zimmer

The corona virus disease 2019 (COVID-19) pandemic, caused by severe acute respiratory syndrome coronavirus 2 (SARS-CoV-2), has become a big threat to human health. Studies have shown that SARS-CoV-2 can also invade the central nervous system (CNS) [1]. Currently, it remains unclear if SARS-CoV-2 infection could cause status epilepticus [2]. However, some viruses or their sub-classes have been known to cause brain damage, which would then initiate or worsen seizure attacks (Table 1). A comprehensive understanding of the relationship between viral infection and seizure would provide insight into the effects of SARS-CoV-2 on seizures, in this particular COVID-19 crisis. In this special issue, we provide a collection of papers discussing on the following questions: which viruses can induce epileptic seizures? In what way do viruses invade human body? What are the vectors for different viruses? Is seizure directly caused by a virus or indirectly induced by immune response? What are the seizure features and electroencephalogram findings after virus infection? What is the treatment and prognosis for viral infection? Can we develop specific vaccines for viral infection?

**Table 1:** The typical viruses associated with seizures and features of resulting seizures in humans or animal models

Virus family	Typical seizure related virus	Seizure features
RNA virus:		
*Flaviviridae*	West Nile virus, Japanese encephalitis virus, St Louis Encephalitis virus, Dengue viruses (types 1–4), Tick-borne encephalitis virus	Generalized tonic-clonic seizures; Partial motor with secondary generalization; Non-convulsive and convulsive status epilepticus [3]; Acute flaccid paralysis [4]
	Zika virus	Early and refractory epilepsy [5]
*Bunyaviridae*	La Crosse virus	Complex partial or simple partial focal seizure; Periodic epileptiform discharges [6]
*Orthomyxoviridae*	Influenza virus	Generalized tonic-clonic seizures; Convulsive status epilepticus [3]; Febrile seizures [7]
*Paramyxoviridae*	Mumps virus	Generalized tonic-clonic seizures [3]; Acute flaccid paralysis [4]
	Measles virus, Canine distemper virus	Generalized tonic-clonic seizures [3]
	Nipah virus	Convulsive status seizures [8]
*Rhabdoviridae*	Rabies virus	Generalized tonic-clonic seizures [3]; Febrile seizure [9]; Focal facial and limb seizures [10]; Acute flaccid paralysis [4]
	Chandipura virus	Generalized tonic-clonic seizures; Focal seizure [11]
*Retroviridae*	Human immunodeficiency virus	Generalized tonic-clonic seizures; Partial motor with/without secondary generalization; Atonic seizures; Non-convulsive and convulsive status epilepticus; High incidence of recurrence [3]
*Picornaviridae*	Enterovirus 70	Generalized tonic-clonic seizures [3] Complex partial or simple partial focal seizure [6] Febrile seizure [12]
	Enterovirus 71	Complex partial or simple partial focal seizure [6] Febrile seizure [12]
	Poliovirus	Acute flaccid paralysis [4]
	Rhinovirus	Febrile seizure [12]
*Togaviridae*	Eastern equine encephalitis viruses	Complex partial or simple partial focal seizure; Periodic epileptiform discharges [6]
*Reoviridae*	Rotavirus	Febrile seizures [13]
*Paramyxoviridae*	Human parainfluenza viruses	Febrile seizures [13]
*Coronaviridae*	Severe acute respiratory syndrome coronavirus	Abnormity located in the temporal lobe accompanied with seizures [14]; Refractory status epilepticus [15]
	Middle East respiratory syndrome–related coronavirus, Human coronavirus OC43, Human coronavirus HKU1	Febrile seizures [16]
	Severe acute respiratory syndrome coronavirus 2	Focal seizure [17]
*Pneumoviridae*	Respiratory syncytial virus/orthopneumovirus	Febrile seizure [12]; Status epilepticus [15]
	Human metapneumovirus	Focal seizure; Status epilepticus [15]; Febrile seizures [13]
*Caliciviridae*	Norovirus	Febrile seizures [13]
*Astroviridae*	Astrovirus	Febrile seizures [13]
DNA virus:		
*Herpesviridae*	Herpes simplex type 1, Herpes simplex type 2	Generalized tonic-clonic seizures; Complex partial seizures; Non-convulsive and convulsive status epilepticus [3]; Periodic epileptiform discharges; Complex partial or simple partial focal seizure [6]
	Human herpes virus 6	Generalized tonic-clonic seizures [3]; Febrile seizures and hippocampal sclerosis [18]; Often partial seizures, prolonged seizures, and repeated seizures [19]
	Cytomegalovirus	Generalized tonic-clonic Seizures; Complex partial seizures; Non-convulsive and convulsive status epilepticus [3]
	Epstein-Barr virus	Generalized tonic-clonic seizures; Complex partial seizures; Non-convulsive and convulsive status epilepticus [3]
*Adenoviridae*	Adenovirus	Acute flaccid paralysis [4] Febrile seizure [12]
*Parvoviridae*	Bocaparvovirus	Febrile seizure [12]

*Acta Epileptologica* is an international academic journal that publishes advances in epileptic research. Viruses are an important cause for seizures. To highlight the important roles of viruses in epilepsy, especially under the current COVID-19 pandemic, *Acta Epileptologica* publishes this special issue on “Virus and Epilepsy”, focusing on four viruses: arbovirus, human immunodeficiency virus (HIV), picornavirus, and human herpes virus 6 (HHV6), from perspectives of pathogenesis, pathogenicity, clinical characteristics, treatment, and prognosis of seizure induction or enhancement after viral infection. Most importantly, the mechanisms underlying viral association with epileptogenesis are also discussed.

In the paper entitled “*Arbovirus and seizures*”, Mingrui Zheng et al. summarized the most common arboviruses associated with epidemic viral encephalitis. Viral encephalitis affects both children and adults, usually leading to severe neurological sequelae. Arboviruses of the genus *Flavivirus* are usually transmitted by mosquitoes and other host animals. Among various arboviruses, the Japanese encephalitis virus, West Nile virus, Zika virus, Dengue virus and Chikungunya virus can induce seizures. Seizures may not be the major manifestation, but may predict a poor prognosis. The occurrence of seizure is mainly caused by direct cell damage by the virus, secondary glial and immune responses producing inflammatory cytokines, and damage to the blood-brain barrier. Different virus infections may lead to mild or severe symptoms, with varied occurrence of epilepsy. The seizure type can usually be focal or generalized, or even status epilepticus. The generalized tonic-clonic seizures are the most common type. EEG recordings have consistently found a pattern of diffuse slow activity. During occurrence of seizure, patients typically manifest with abnormalities including theta and delta coma, burst suppression, an isoelectric pattern, or occasionally alpha coma. Effective treatment of associated epileptic seizures, mostly symptomatic support and anti-virus therapy, enables good supportive care and optimal control of CNS-related comorbidities. However, usually there is a lack of effective treatment methods.(https://aepi.biomedcentral.com/articles/10.1186/s42494-020-00026-w).

In the paper entitled “*The role of picornavirus infection in epileptogenesis*”, Runxuan Zhang et al. summarized the clinical characteristics of picornavirus infection, and the pathogenesis of Theiler’s murine encephalomyelitis virus (TMEV)-induced epilepsy. Picornaviruses are a family of small positive-strand RNA viruses and transmitted via the respiratory or fecal-oral route. The neurotropic picornaviruses can induce acute or late recurrent seizures following CNS infection, by infecting the peripheral nerve, crossing the blood-brain barrier and migrating in the Trojan-horse approach. TMEV, as a member of the *Picornavirus* family, can cause encephalitis, leading to chronic spontaneous seizures. The TMEV-infected animal model has been used to study the mechanisms of epileptogenesis and to evaluate drug efficacy. Several immune components are involved in TMEV-induced epileptogenesis. Minocycline (MIN) treatment can positively affect the long-term epileptogenic process. Valproic acid has a neuroprotection effect through inhibition of histone deacetylase activity, thereby reducing seizures. Conversely, Carbamazepine may exacerbate TMEV infection, making animals more susceptible to the virus. Wogonin can decrease epileptic episodes by inhibiting the activated macrophages targeted by the IL-6 producing cells in mice. (https://aepi.biomedcentral.com/articles/10.1186/s42494-021-00040-6).

HHV-6 is a ubiquitous and most common pathogen that affects humans. In the paper entitled “*Temporal lobe epilepsy associated with human herpes virus 6*”, Jiaqi Wang et al. summarized the close relationship between HHV-6B and temporal lobe epilepsy (TLE). The special target of HHV-6 is in the hippocampus and amygdala. Recently, viral genomic DNA of HHV-6B has been detected in surgically removed brain tissues of intractable epilepsy patients, suggesting the involvement of HHV-6B in the pathogenesis of epilepsy. TLE patients with HHV-6B in their brains suffer from reiterative attacks of febrile seizures and hippocampal sclerosis. In this article, the potential involvement of cytokines in the pathological process of TLE is reviewed in detail. Anti-inflammatory or immunomodulatory therapies may show prospects for TLE treatment. (https://aepi.biomedcentral.com/articles/10.1186/s42494-021-00044-2).

The article entitled “*Current epidemiological and etiological characteristics and treatment of seizures or epilepsy in patients with HIV infection*” by Changhao Yu et al. carefully reviewed the role of HIV infection in epilepsy. Opportunistic infections are a stereotypical predisposing factor for seizures in HIV patients. There is a prevalence of 2–19.8% and an incidence of 1.8–19.8% for seizures in the HIV-infected population, which are mostly of the generalized type. HIV patients with seizures need to take both antiviral and antiepileptic drugs, which increases the risk of drug-drug interactions and the occurrence of side effects. Antiepileptic drugs should be carefully selected to avoid the CYP450-induced drug-drug interactions. This review further gives a perspective on future therapeutic studies in HIV patients with seizures. (https://aepi.biomedcentral.com/articles/10.1186/s42494-020-00028-8).

## Data Availability

Not applicable.

## Abbreviations

CNS: Central nervous system
COVID-19: Corona virus disease 2019
SARS-CoV-2: Severe acute respiratory syndrome coronavirus 2
EEG: Electroencephalogram
TMEV: Theiler’s murine encephalomyelitis virus
MIN: Minocycline
VPA: Valproic acid
HDAC: Histone deacetylase
HHV-6: Human herpes virus 6
TLE: Temporal lobe epilepsy
HIV: Human immunodeficiency virus
CBZ: Carbamazepine
